# Adverse events associated with benznidazole treatment for Chagas disease in children and adults

**DOI:** 10.1111/bcp.16214

**Published:** 2024-08-29

**Authors:** Cintia Valeria Cruz, Andres Rabinovich, Guillermo Moscatelli, Samanta Moroni, Nicolas González, Facundo Garcia‐Bournissen, Griselda Ballering, Hector Freilij, Jaime Altcheh

**Affiliations:** ^1^ Servicio de Parasitología y Instituto Multidisciplinario de Investigación en Patologías Pediátricas (IMIPP), CONICET‐GCBA Hospital de Niños Ricardo Gutierrez Buenos Aires Argentina; ^2^ Mahidol Oxford Research Unit (MORU) Centre for Tropical Medicine and Global Health, Nuffield Department of Medicine, University of Oxford, Oxford, UK; ^3^ Division of Paediatric Clinical Pharmacology, Department of Paediatrics, Schulich School of Medicine and Dentistry Western University London Ontario Canada

**Keywords:** adults, adverse drug reactions, benznidazole, Chagas disease, children, congenital, *Trypanosoma cruzi*

## Abstract

**Aims:**

Chagas disease (ChD) affects approximately 7 million people in Latin America, with benznidazole being the most commonly used treatment.

**Methods:**

Data from a retrospective cohort study in Argentina, covering January 1980 to July 2019, was reanalysed to identify and characterize benznidazole‐related adverse drug reactions (ADRs).

**Results:**

The study included 518 patients: 449 children and 69 adults (median age in children: 4 years; adults: 25 years; age ranges: 1 month–17.75 years and 18–59 years, respectively). The median benznidazole doses received were 6.6 mg/kg/day for at least 60 days in children and 5.6 mg/kg/day for a median of 31 days in adults. Overall, 29.34% (152/518) of patients developed benznidazole‐related ADRs, with an incidence of 25.83% (116/449) in children and 52.17% (36/69) in adults (odds ratio [OR] = 0.32, 95% confidence interval [CI] = 0.19–0.54, *P* < .001). The incidence rate was 177 cases per 1000 person‐years (95% CI = 145–214) in children and 537 per 1000 person‐years (95% CI = 360–771) in adults. There were 240 ADRs identified, primarily mild to moderate. Severe ADRs occurred in 1.11% (5/449) of children and 1.45% (1/69) of adults. The skin was the most affected system. A total of 10.23% (53/518) of patients discontinued treatment. More adults than children discontinued treatment (OR = 3.36, 95% CI = 1.7–6.4, *P* < .001).

**Conclusions:**

Although 29.34% of patients experienced ADRs, most were mild to moderate, indicating a manageable safety profile for benznidazole. While optimized dosing schedules and new drugs are needed, avoiding benznidazole solely due to safety concerns is not justified.

**What is already known about this subject**

- Chagas disease, a neglected tropical disease affecting >7 million people globally, is commonly treated with benznidazole.
- Prescriber hesitancy arises from unpredictable drug availability, requirement for long treatment periods, unclear pharmacokinetic‐pharmacodynamic (PKPD) profile and a perceived high incidence of severe adverse events, which is not well supported by the evidence.

**What this study adds**

- From 1980 to 2019, 152 out of 518 individuals (29.34%) developed ADRs, slightly lower than previously reported.
- Benznidazole‐related ADRs were significantly more frequent in adults than in children, with the skin being the most affected system.
- Most ADRs related to benznidazole in our cohort were mild or moderate and resolved without sequelae.

## 1 INTRODUCTION

Chagas disease (ChD) is an infection caused by the kinetoplastid protozoa, *Trypanosoma cruzi* (*T. cruzi*), and is transmitted by various species of hematophagous reduviid insects, commonly known as “kissing bugs”. *T. cruzi* can also be transmitted vertically, by organ donation or blood transfusion, laboratory accidents and orally, through the ingestion of food/drink contaminated with triatomines or their faeces. ChD is a chronic, stigmatizing condition, closely associated with poverty, and remains one of the most neglected among the tropical diseases. It is endemic in 21 Latin American countries and as a result of migration has extended to many other countries around the world.^1^

In the vector‐transmitted form of the disease, almost 90% of patients are asymptomatic during the acute phase. After evolving to the chronic phase, about 20% of patients develop clinical manifestations with the cardiovascular, gastrointestinal and/or nervous systems being the most severely affected.^2^

ChD was described by Carlos Chagas more than 100 years ago and causes significant social and economic burden (particularly in endemic areas). Despite this, surprisingly, only two drugs exist to treat this condition: benznidazole (BZ) and nifurtimox (NFX). However, it is estimated that less than 1% of ChD‐infected patients receive pharmacological treatment.^3^ Both drugs have controversial safety profiles, as the reported frequency of adverse events varies from 0 to 98%.^4^ Nevertheless, BZ and NFX tend to be better tolerated in children than in adults.^5^, ^6^ The World Health Organization (WHO), through Pan American Health Association (PAHO) guidelines,^7^ reflected in the Argentine national guideline,^8^ clearly state that all patients with acute Chagasdisease, and most patients with chronic disease, should receive antiparasitic treatment. Unfortunately, this recommendation is not universally followed, in part due to unpredictable drug availability, the requirement for long treatment periods, an unclear PKPD profile and the frequently claimed high incidence of adverse events, the latter not being well supported by the evidence.

The objective of this paper is to describe and compare the safety of BZ in adult and paediatric patients based on data from a large cohort of ChD patients, including infants, children and adults treated with BZ.

## 2 METHODS

### 2.1 Study design

This was a retrospective age‐stratified study to assess the safety and tolerability of oral BZ in subjects with ChD. All patients were treated and followed up at the Parasitology and Chagas Service, Hospital de Niños “Ricardo Gutiérrez”, Buenos Aires, Argentina, from January 1980 to July 2019. The administered treatments adhered to the contemporary standards of care guidelines for ChD at the time of prescription. The Parasitology and Chagas Service at Hospital de Niños “Ricardo Gutiérrez” serves as a national reference centre located within an urban setting, situated in a tertiary care level paediatric hospital.

In an environment devoid of vectorial transmission, the service primarily receives referrals of newborns born to mothers identified with Chagas disease during the national pregnancy screening programme. In many instances, these newborns serve as the “index cases”, facilitating comprehensive family screening and, once infected relatives are identified, case treatment. This is why the majority of adult patients who visit our site are the mothers of the newborns.

### 2.2 Population

All patients with diagnosis of Chagas disease were eligible for inclusion in the study. The diagnostic criteria used to confirm the diagnosis of ChD were as follows: for infants younger than eight months: direct observation of *T. cruzi* using the parasitological concentration method (microhaematocrit test, MH) or xenodiagnoses (XD); for older patients: two reactive serological tests – enzyme‐linked immunosorbent assay (ELISA), indirect haemagglutination (IHA) or direct agglutination (DA). Exclusion criteria included cases where benznidazole was prescribed but not taken and or patients lost to follow‐up.

Patients were stratified by age, with those 18 years and older considered adults. We substratified paediatric patients by age (0–7 months, 8 months–1 year, 2–6 years, 7–11 years and 12–17 years) based on previous studies showing substantial changes in treatment tolerability in children older than 7 years.^9^, ^10^, ^11^ For the safety analysis, all patients who started treatment were considered, regardless of whether they completed the full treatment course.

### 2.3 Procedures

#### 2.3.1 Treatment

BZ treatment (12.5 mg, 100 mg, Radanil [Roche] or Abarax [ELEA]) was prescribed in doses of 5–8 mg per kg per day divided in two daily doses for 30–60 days, according to national guidelines at the time of diagnosis. (From 2018, a treatment is considered complete if the patient received the drug for 30 days.^8^, ^12^) Enrolment of children started in March 1980, and enrolment of adults started in July 2004. Infant BZ doses were provided as fractionated tablets prepared by a pharmacist and administered with water or mother's milk. Medication was provided to patients or their guardians in monthly batches, and compliance was assessed by counting the remaining tablets at each visit. Treatment was considered complete when patients took the medication for at least 55 days for children and 28 days for adults (≥17 years of age).

#### 2.3.2 Data collection

Data were collected from medical records of treated patients and entered into an Access clinical database designed for this study. All individual datasets were anonymized. Demographic data, clinical and biochemical assessments and complementary studies (haematology, hepatology and renal function biochemical tests, and pregnancy test for females of childbearing potential), were collected during follow‐up. Baseline data were obtained prior to the first dose of treatment. Following the standard of care at the Hospital de Niños “Ricardo Gutiérrez” for ChD‐treated patients, follow‐up visits were carried out at 7 and 30 days, at the end of treatment, every 3 months during the first‐year post‐treatment and then every 6–12 months thereafter. Adverse drug reactions (ADRs) were evaluated through laboratory tests, clinical interviews and physical examinations, and classified according to WHO definitions.^13^, ^14^ Causality assessment was performed using the WHO criteria. Information on treatment duration and dosage, temporary interruptions and concomitant medications were systematically collected from medical records and documented in the clinical database.

#### 2.3.3 Statistical analysis

Mean and median with corresponding standard deviation or interquartile range were used as summary statistics for continuous variables while categorical variables were summarized using percentages. Statistical analysis was carried out using R. A median‐unbiased estimation (mid‐*P*) test (function odds ratio) from the epitools package was used for statistical significance testing of odds ratios. For rates, a proportions test (function prop.test) from the stats package (included in R) was used. The function epi.conf from epiR package was used to estimate incidence rates. *P*‐values < .05 were considered statistically significant.

## 3 RESULTS

### 3.1 Population characteristics

**FIGURE 1:**
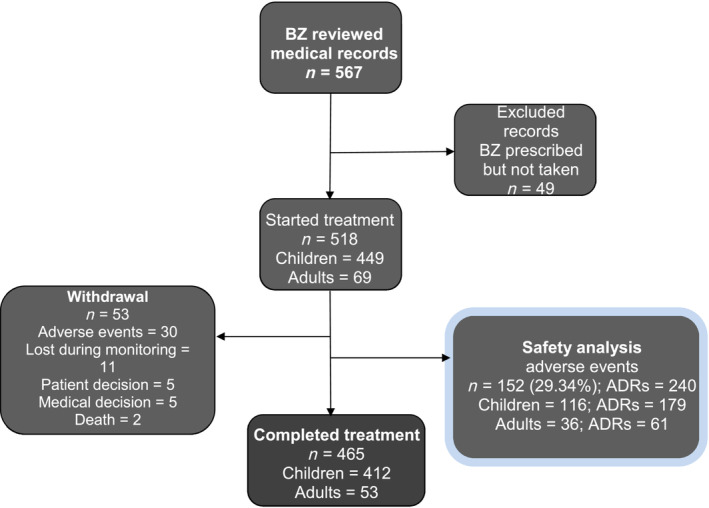
Flowchart of cohort patients.

Medical records of ChD patients treated at the Hospital de Niños “Ricardo Gutiérrez” were reviewed, and 567 patients who were prescribed BZ were identified. After excluding patients who did not start treatment, 518 patients were included in the study: 449 children (age range: 1 month–17.75 years) and 69 adults (Figure 1). The age range of the adults included was 18–59 years, with a median of 25 years (interquartile range [IQR]_25–75_ = 20–34). Among the children, 16.03% (72/449) were 0–7 months old, 22.94% (103/449) were 8 months–1 year old, 19.82% (89/449) were 2–6 years old, 20.71% (93/449) were 7–11 years old, and 20.49% (92/449) were 12–17 years old (Table 1).

**TABLE 1:** Baseline demographic data of patients included in the study.

	Children (%)	Adults (%)	Total patients (%)
**Gender**			
Female	225 (50.11)	54 (78.26)	279 (53.86)
Male	224 (49.89)	15 (21.74)	239 (46.14)
**Age**			
Median [Q1, Q3]	48 (10, 132) months	25 (20, 34) years	‐
Mean (SD)	70.33 (64.87) months	27.46 (9.29) years	‐
Min‐Max	1–213 months	18–59 years	‐
**Route of infection**			
Vector	20 (4.45)	9 (13.04)	29 (5.6)
Congenital	280 (62.36)	14 (20.29)	294 (56.75)
Blood transfusion	2 (0.22)	1 (1.45)	3 (0.58)
Undetermined	147 (32.74)	45 (65.21)	192 (37.06)
**Clinical examination at diagnosis**			
Asymptomatic	411 (91.53)	68 (98.55)	479 (92.47)
Symptomatic	38 (8.46)	1 (1.45)	39 (7.53)
**Total**	**449 (100)**	**69 (100)**	518 (100)

Most patients were asymptomatic at diagnosis: 97.1% (436/449) were children and 98.55% (68/69) were adults (Table 2). The majority of symptomatic cases, 78% (11/14), were in children in the age group 0–8 months. The most frequent sign detected was jaundice, in 28.57% (4/14) of cases, and hepatomegaly, in 28.57% (4/14) of cases. Except for two premature babies who died due to complications arising from preterm birth, possibly triggered by congenital ChD infection, all symptomatic patients showed clinical improvement after receiving treatment. Only one symptomatic adult patient contracted ChD via a vector. A detailed case report was published by Bisio et al.^15^

**TABLE 2:** Clinical findings in patients with symptomatic Chagas disease classified by route of infection.

Route of infection/clinical finding	No. of patients (%)
**Vector‐borne**	***n* = 29**
Clinical examination	
Symptomatic	4 (13.8)
Symptoms	5
Chagoma	1 (0.2)
Meningeal syndrome, facial paralysis and paresthesia	1 (0.2)
Cerebral chagoma in CT scan	1 (0.2)
Low weight	1 (0.2)
Splenomegaly	1 (0.2)
**Congenital**	**n = 294**
Clinical examination	
Symptomatic	10 (3.4)
Symptoms	13
Jaundice	4 (30.77)
Neonatal hepatitis	3 (23.08)
Hepatomegaly	4 (30.77)
Splenomegaly	2 (15.38)

*Note*: All patients infected by undetermined routes were asymptomatic. Note that a patient may present with more than one symptom.

A median dose of BZ of 6.6 mg/kg/day (IQR_25–75_ = 5.7–7.3) was received for at least 60 days by 84.18% (378/449) of children. Adult patients received a median BZ dose of 5.6 mg/kg/day (IQR_25–75_ = 5.2–6.1) for a median duration of 31 days (IQR_25–75_ = 30–60) (Table 7).

Only 10.23% (53/518) of patients discontinued treatment (Figure 1) after a mean of 14 days (IQR_25–75_ = 10–20). The number of male and female children in the study was very similar in contrast to 78.26% (54/69) of all adult subjects being female (most of whom were mothers of children followed up at the Hospital de Niños “Ricardo Gutiérrez”). Congenitally acquired infection occurred in 62.36% (280/449) of the children whilst only 20.29% (14/69) of adults were confirmed as congenitally acquired cases. The other routes of infection identified were: undetermined in 32.74% (147/449) of children and 65.21% (45/69) of adults, through a vector in 4.45% (20/449) of children and 13.04% (9/69) of adults, and blood transfusion in 0.44% (2/449) of children and 1.45% (1/69) of adults (Table 1).

### 3.2 Incidence of ADRs

**FIGURE 2:**
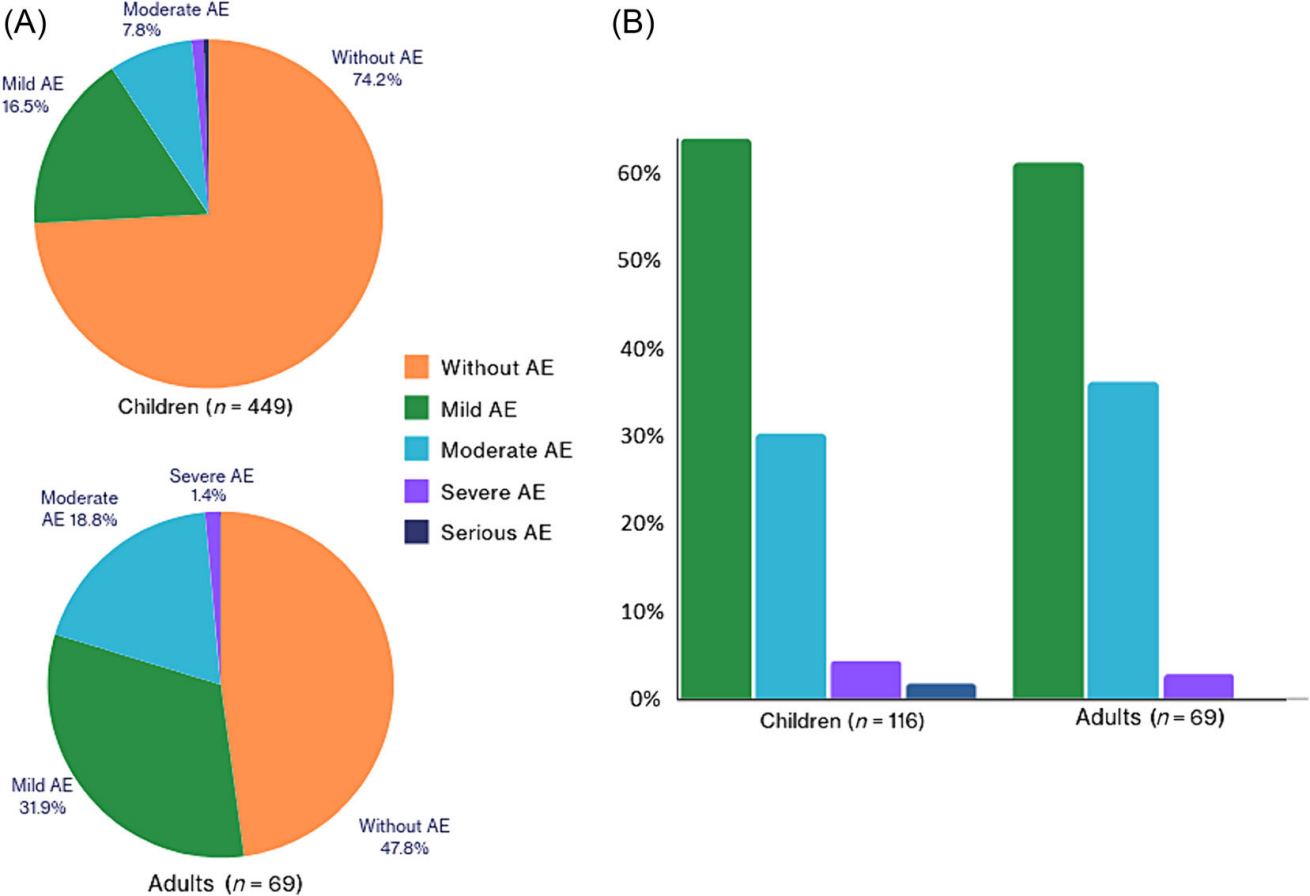
BZ‐related ADRs. (A) Proportion of patients affected by ADRs. (B) Classification of ADRs by severity (in the 152 patients affected).

In total, 35.9% (186/518) of patients experienced adverse events, with an incidence of 32.96% (148/449) in children and 55.07% (38/69) in adults, and the resulting global OR = 0.4, 95% CI = 0.24–0.67; *P* < .001. Overall, 29.34% (152/518) of patients developed BZ‐related ADRs, with an incidence of 25.83% (116/449) in children and 52.17% (36/69) in adults (OR = 0.32, 95% CI = 0.19–0.54, *P* < .001) and an incidence rate of 177 cases per 1,000 person‐years (95% CI = 145–214) and 537 cases per 1000 person‐years (95% CI = 360–771) in adults (Figure 2).

A total of 311 ADRs were documented, with 243 occurring in children (median of 1 ADR per patient, IQR_25–75_ = 1–2) and 68 in adults (median 1 ADR per patient, IQR_25‐75_ = 1–2). Out of the total of 311 ADRs, 240 were deemed to be BZ‐related. BZ‐related ADRs were less frequent in children, constituting 73.66% (179/243) of events, compared to adults, where 89.7% (61/68) of events were observed (OR = 0.32, 95% CI = 0.13–0.7; *P* < .01) (Table 4 and 5).

**FIGURE 3:**
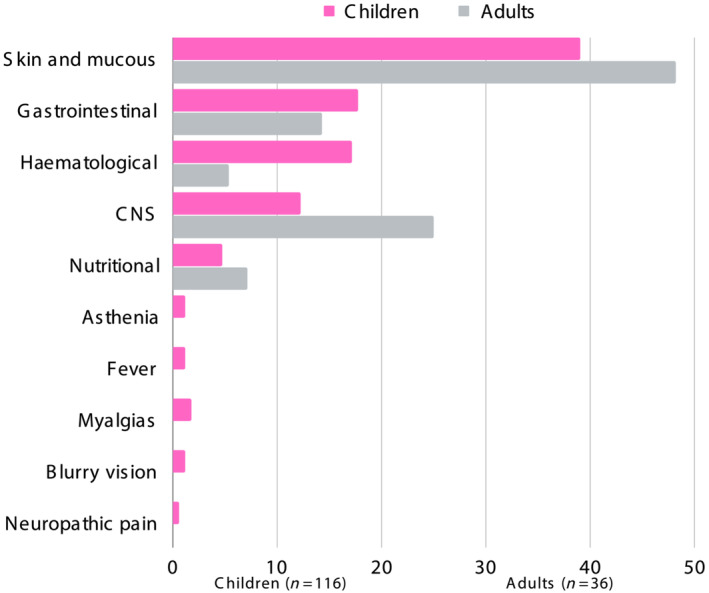
Classification of the 240 BZ‐related ADRs per organ/system affected in each population (adults *vs.* children).

Cutaneous and mucosal tissues were the most commonly affected systems in both children (39.66% of events, 71/179 events) and adults (52.45% of events, 32/61 events). In children, next most common was gastrointestinal system involvement (21.22% of events, 38/179 events) followed by haematological system involvement (mostly anaemia and neutropenia), which accounted for 16.76% of events (30/179 events). In adults, headaches (the only ADR affecting the central nervous system [CNS]) accounted for 22.95% of events (14/61 events) whilst adverse events involving the gastrointestinal system account for 13.11% of events (8/61 events). Mild myalgia, without associated creatine phosphokinase elevation, and blurred vision were observed only in children. A detailed description of the 240 BZ‐related ADRs events are shown in Table 5 and Figure 3.

The most common skin manifestation was pruriginous rash or urticaria (Table 5 and Figure 3). In all cases, BZ administration was stopped and symptomatic treatment (antihistamines and, in some patients, glucocorticoids) was administered until the skin manifestations resolved. Median time to resolution was 5 days (IQR_25–75_ = 2–10).

Thirteen patients developed neutropenia (total neutrophil count < 1500). This was mild in all cases, except for four patients, where it was moderate (total neutrophil count < 1000). Among these four patients with moderate neutropenia, one also developed anaemia, as evidenced by a decrease in haemoglobin levels from 12.6 mg/dL to 9.1 mg/dL over a 1‐month interval. Only one patient discontinued treatment due to haematologic ADRs, and in all affected patients, neutropenia and anaemia resolved completely within approximately 1 month.

Seven children experienced hepatic ADRs, characterized by elevated transaminase levels. No increase in bilirubin was observed for these patients. No adults experienced hepatic ADRs.

**FIGURE 4:**
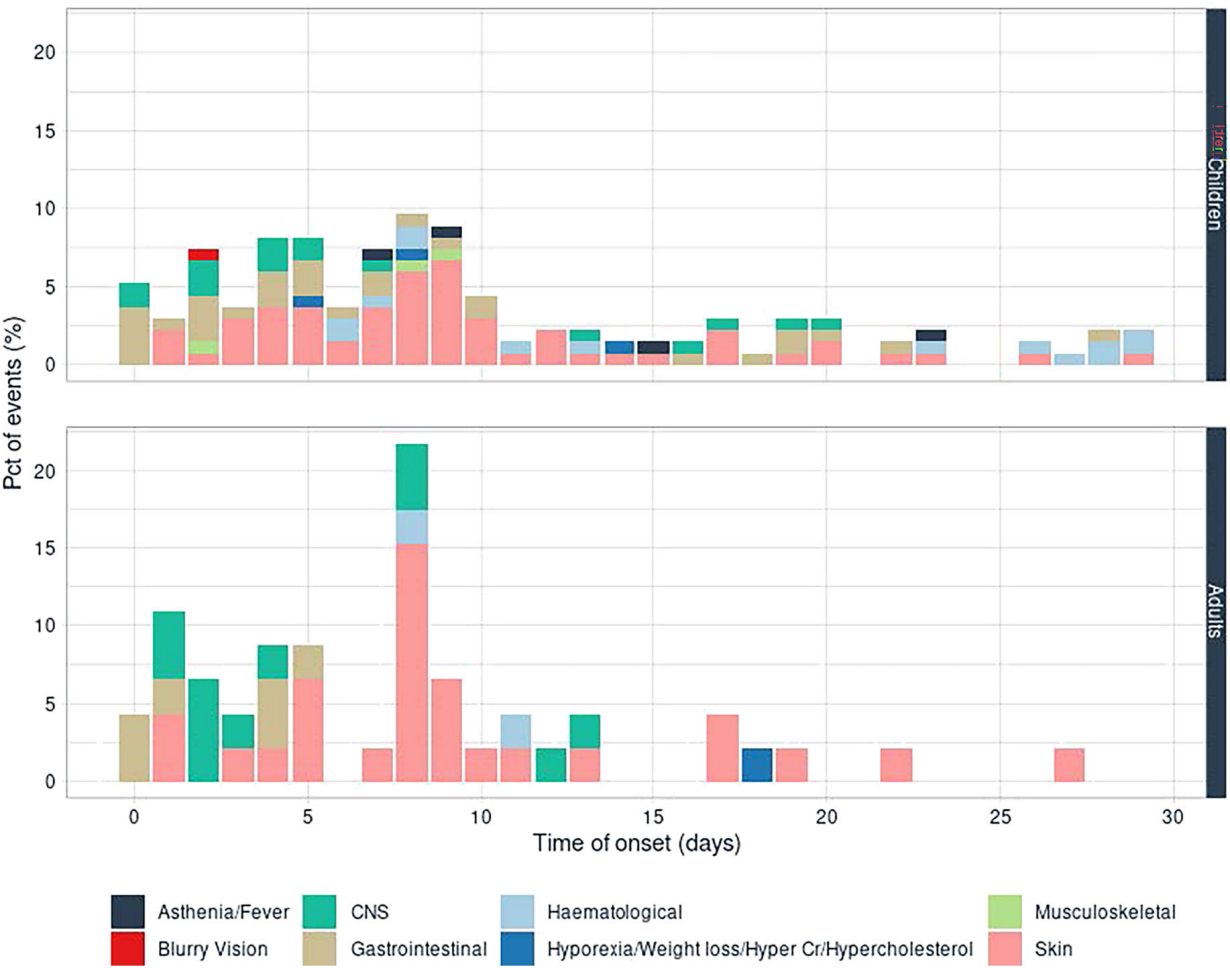
Time of onset in days for benznidazol‐related ADRs categorized by organ or system affected.

Time to onset of ADRs was recorded for 93.75% (225/240) of BZ‐related ADRs and for at least one event for 143 patients, with 78.22% (176/225) of ADRs appearing within 30 days of treatment. The median onset time of ADRs was 9 days (IQR_25–75_ = 5.25–14.75) for skin, 5 days (IQR_25–75_ = 2–16) for gastrointestinal, 39 days (IQR_25–75_ = 14–62) for nutritional, and 4 days (IQR_25–75_ = 2–13) for CNS ADRs (Figure 4).

### 3.3 Severity of ADRs

The majority, 72.5% (174/240), of the 240 documented BZ‐associated ADRs manifested were mild and 24.58% (59/240) were moderate in severity and resolved without sequelae. Severe ADRs were observed in 1.15% (6/518) of patients. One adult patient experienced a non‐specific rash and discontinued treatment, while two children developed rashes that led to stopping the drug, one by medical indication and the other by parents' decision. Additionally, serious ADRs were observed in 0.44% (2/449) of children: one patient fulfilled clinical and laboratory criteria for DRESS (drug reaction with eosinophilia and systemic symptoms), and the other exhibited fever and generalized exanthema. In both of these cases, patients were admitted to the paediatric ward and the treatment was discontinued by medical decision. All the severe and serious ADRs resolved without sequelae. The severity of ADRs and their relationship with BZ are described in Table 3 and Figure 2.

**TABLE 3:** Treatment description.

	Children	Adults	Total patients
**Dose (**mg/kg body weight)			
Median [Q1, Q3]	6.6 [5.7, 7.3]	5.6 [5.2, 6.1]	6.4 [5.6, 7.2]
Missing (%)	14 (3.4)	‐	14 (3.01)
**Number of doses**			
Median [Q1, Q3]	2 [2, 2]	2 [2, 2]	2 [2, 2]
Missing (%)	10 (2.42)	‐	10 (2.15)
**Days of treatment**			
Median [Q1, Q3]	60 [60, 60]	31 [30, 60]	60.0 [60.0, 60.0]
Missing (%)	5 (1.21)	‐	5 (1.07)
**Concomitant medication**			
Yes (%)	30 (7.28)	1 (1.88)	31 (6.7)
No (%)	382 (92.72)	52 (98.11)	434 (93.33)
**Compliance**			
Yes (%)	350 (84.95)	45 (84.9)	395 (84.94)
No (%)	62 (15.05)	8 (15.09)	70 (15.05)
**Temporary interruption**			
Yes (%)	51 (12.37)	4 (7.54)	55 (11.82)
No (%)	361 (87.62)	49 (92.45)	410 (88.17)
**Total**	**412 (88.6)**	**53 (11.4)**	**465 (100)**

*Note*: Treatment dosing, length, compliance and presence of concomitant medication for all patients that completed treatment with benznidazole (*n* = 465).

**TABLE 4:** Number and percentage of all adverse events in the cohort classified by severity and their relationship to treatment.

	No. (%) among:			
	Children		Adults	
ADR classification	Patients with ADRs	No. of ADRs	Patients with ADRs	No. of ADRs
	(*n* = 148)	(*n* = 243)	(*n* = 38)	(*n* = 68)
**Severity**				
Mild	120 (70.59)	184 (75.72)	32 (66.67)	48 (70.59)
Moderate	38 (22.35)	46 (18.93)	15 (31.25)	19 (27.94)
Severe	9 (5.29)	9 (3.7)	1 (2.08)	1 (1.47)
Serious	2 (1.78)	3 (1.23)	0 (0)	0 (0)
No data	1 (0.59)	1 (0.41)	0 (0)	0 (0)333
**Total**	**170**	**243**	**48**	**68**
**Relationship to treatment**				
None	46 (24.73)	56 (23.45)	2 (4.35)	4 (5.88)
Unlikely	7 (3.76)	7 (2.88)	2 (4.35)	2 (2.94)
Probable	79 (42.47)	111 (45.68)	20 (43.48)	27 (39.7)
Certain	53 (23.12)	68 (27.98)	21 (45.65)	34 (50)
No data	1 (0.55)	1 (0.41)	1 (2.17)	1 (1.47)
**Total**	**186**	**243**	**46**	**68**

*Note*: Relationship classification was recorded according to OMS criteria. For each age group, two columns are shown. The first one, *Patients with ADRs*, shows the number of patients presenting with at least one ADR and its corresponding percentage. The second column, *Number of ADRs*, depicts the observed number of ADRs and its corresponding percentage. Notice that patients could present with more than one ADR belonging to different categories (i.e Mild–Moderate).

**TABLE 5:** BZ‐related adverse reaction occurrence and incidence by organ/system.

	Children		Adults	
	Patients with ADRs (%)	Number of ADRs (%)	Patients with ADRS (%)	Number of ADRs (%)
	*n* = 116	*n* = 179	*n* = 36	*n* = 61
**Skin and mucous**	**66 (39.05)**	**71 (39.66)**	**27 (48.21)**	**32 (52.46)**
Exanthema (pruriginous rash)	44 (66.67)	49 (69.01)	23 (85.19)	28 (87.5)
Urticaria/angioedema	10 (15.15)	10 (14.08)	3 (11.11)	3 (9.375)
Eczema	9 (13.64)	9 (12.68)	1 (3.7)	1 (3.125)
Exanthema (petechiae rash)	1 (1.51)	1 (1.4)	‐	‐
Polymorphous erythema	1 (1.51)	1 (1.4)	‐	‐
DRESS	1 (1.51)	1 (1.4)	‐	‐
**Gastrointestinal**	**35 (17.75)**	**38 (21.23)**	**8 (14.29)**	**8 (1.31)**
Increased hepatic enzymes	12 (34.29)	12 (31.58)	‐	‐
Abdominal pain	10 (28.57)	10 (26.32)	2 (25)	2 (25)
Nausea and vomiting	9 (25.71)	12 (31.58)	5 (62.5)	5 (62.5)
Diarrhoea	2 (5.71)	2 (5.26)	1 (12.5)	1 (12.5)
Constipation	1 (2.86)	1 (2.63)	‐	‐
Spasms	1 (2.86)	1 (2.63)	‐	‐
**Haematological**	**29 (17.16)**	**30 (16.76)**	**3 (5.36)**	**3 (4.92)**
Leukopenia	14 (48.28)	15 (50)	2 (66.67)	2 (66.67)
Eosinophilia	14 (48.28)	14 (46.67)	1 (33.33)	1 (33.33)
Leukocytosis	1 (3.45)	1 (3.33)	‐	‐
**Central Nervous System (CNS)**	**21 (12.42)**	**22 (12.29)**	**14 (25)**	**14 (22.95)**
Headache	16 (76.19)	17 (77.27)	13 (92.86)	13 (92.86)
Dizziness	4 (19.05)	4 (18.18)	1 (7.14)	1 (7.14)
Irritability	1 (4.76)	1 (4.54)	‐	‐
**Nutritional**	**8 (4.73)**	**8 (4.47)**	**4 (7.14)**	**4 (6.56)**
Hyporexia	5 (62.5)	5 (62.5)	1 (25)	1 (25)
Weight loss	1 (12.5)	1 (12.5)	3 (75)	3 (75)
Creatinine elevation	1 (12.5)	1 (12.5)	‐	‐
Hypercholesterolemia	1 (12.5)	1 (12.5)	‐	‐
**Body as a whole**	**4 (2.37)**	**4 (2.23)**	‐	‐
Asthenia	2 (50)	2 (50)	‐	‐
Fever	2 (50)	2 (50)	‐	‐
**Myalgias**	**3 (1.77)**	**3 (1.68)**	‐	‐
**Blurry vision**	**2 (1.18)**	**2 (1.12)**	‐	‐
**Neuropathic pain in hands and feet**	**1 (0.59)**	**1 (0.56)**	‐	‐
**Total**	**169 (100)**	**179 (100)**	**56 (100)**	**61 (100)**

*Note*: Detailed description of the 240 BZ‐related ADRs occurring in the 152 patients segregated by organ system. Note that a patient may present with more than one ADR. For each age group, two columns are shown. The first column, *Patients with ADRs*, shows the number of patients presenting with at least one ADR and its corresponding percentage. The second column, *Number of ADRs*, describes the observed number of ADRs and its corresponding percentage.

**TABLE 6:** Treatment discontinuation and interruption.

	Children (%)	Adults (%)	Total patients (%)
**Complete treatment**			
Yes	412 (91.76)	53 (76.81)	465 (89.77)
No	37 (8.24)	16 (23.19)	53 (10.23)
**Discontinuation cause**			
Patient decision	3 (8.11)	2 (12.5)	5 (9.43)
Medical decision	4 (10.81)	1 (6.25)	5 (9.43)
Adverse effect	20 (54.05)	10 (62.5)	30 (56.6)
Death	2 (5.41)	0 (0)	2 (3.77)
Lost to follow‐up	8 (21.62)	3 (18.75)	11 (20.75)
**Temporary interruption**			
Yes	51 (12.38)	4 (7.55)	55 (11.83)
No	361 (87.62)	49 (92.45)	410 (88.17)
**Total**	449 (100)	69 (100)	518 (100)

*Note*: Detailed description of reasons for treatment discontinuation and interruption for all patients included in this study (*n* = 518 patients).

**TABLE 7:** ADRs causing treatment discontinuation.

	Age range (years)	Gender	Symptoms	Treatment length (days)	Second treatment
**Paediatrics**	0–8 months	F	Vomiting, Abdominal pain	21	NF, completed
	0–8 months	F	Leukopenia	39	–
	8 months –2 years	M	Hepatotoxicity	40	–
	8 months –2 years	F	Urticaria/angioedema	20	NF, discontinued by patient decision
	8 months –2 years	M	Exanthema (rash petechiae), Exanthema (pruriginous rash)	18	NF, completed
	2–7	F	Urticaria/angioedema	23	NF, completed
	2–7	F	Exanthema (pruriginous rash), Fever	10	NF, completed
	2–7	M	Exanthema (pruriginous rash)	15	NF, completed
	2–7	M	Exanthema (pruriginous rash), Increased hepatic enzymes	8	–
	7–12	M	Urticaria/angioedema	10	NF, completed
	7–12	M	Exanthema (pruriginous rash)	26	NF, completed
	7–12	M	Exanthema (pruriginous rash)	10	–
	12–18	F	Exanthema (pruriginous rash)	14	–
	12–18	M	Headache	6	–
	12–18	M	Exanthema (pruriginous rash), Headache	15	NF, completed
	12–18	F	Exanthema (pruriginous rash)	21	NF, completed
	12–18	F	Exanthema (pruriginous rash)	11	NF, completed
	12–18	M	Exanthema (pruriginous rash), Eosinophilia	9	NF, completed
	12–18	F	Exanthema (pruriginous rash)	17	NF, completed
**Adults**	18	F	Exanthema (pruriginous rash), Headache	13	NF, completed
	18	F	Exanthema (pruriginous rash)	9	NF, completed
	18	F	Exanthema (pruriginous rash)	10	NF, completed
	19	F	Exanthema (pruriginous rash)	9	NF, discontinued without information
	20	F	Headache, Vomiting, Exanthema (pruriginous rash)	22	–
	24	F	Exanthema (pruriginous rash)	8	NF, completed
	26	F	Weight loss, Exanthema (pruriginous rash)	8	NF, completed
	30	F	Abdominal pain, Headache	12	NF, completed
	37	F	Exanthema (pruriginous rash), Urticaria/angioedema	18	–
	42	F	Headache, Nausea, Exanthema (pruriginous rash)	16	–

*Note*: Detailed ADR description for those patients who discontinued treatment due to ADRs.

Abbreviations: BZ: benznidazol, NF: Nifurtimox. All patients had a good response to symptomatic treatment.

**TABLE 8:** Treatment details for the paediatric cohort.

	(0–7 months)	(8 months–1 year)	(2–6 years)	(7–11 years)	(12–17 years)	Total
	Patients (%)	Patients (%)	Patients (%)	Patients (%)	Patients (%)	Patients (%)
	*n* = 72	*n* = 103	*n* = 89	*n* = 93	*n* = 92	*n* = 449
**Complete treatment**						
Yes	66 (91.67)	99 (96.12)	84 (94.38)	85 (91.4)	78 (84.78)	412 (91.76)
No	6 (8.33)	4 (3.88)	5 (5.62)	8 (8.6)	14 (15.22)	37 (8.24)
**Treatment discontinuation**						
Patient decision	–	1 (25)	–	2 (25)	–	3 (8.11)
Medical decision	–	–	1 (20)	1 (12.5)	2 (14.29)	4 (10.81)
Adverse event	3 (50)	3 (75)	4 (80)	3 (37.5)	7 (50)	20 (54.05)
Death	2 (33.33)	–	–	–	–	2 (5.4)
Loss of follow‐up	1 (16.67)	–	–	2 (25)	5 (35.71)	8 (21.62)
**Temporary interruption**						
Yes	7 (10.6)	12 (12.12)	4 (4.76)	15 (17.65)	13 (16.67)	51 (12.38)
No	59 (89.4)	87 (87.88)	80 (95.24)	70 (82.35)	65 (83.33)	361 (87.62)
**Compliance**						
Yes	59 (89.4)	89 (89.90)	71 (84.52)	70 (82.35)	61 (78.21)	350 (84.95)
No	7 (10.6)	10 (10.1)	13 (15.48)	15 (17.65)	17 (21.79)	62 (15.05)
**Adverse related events**						
Yes	11 (15.28)	19 (19.19)	17 (20.24)	32 (34.41)	37 (40.22)	116 (25.84)
No	61 (84.72)	84 (84.84)	72 (85.71)	61 (65.59)	55 (59.78)	333 (74.16)
**Number of events per patient**						
Median [Q1, Q3]	1 [1, 2]	1 [1, 1.5]	1 [1, 2]	1 [1, 2]	1 [1, 2]	1 [1, 2]

*Note*: Treatment completion, compliance, interruption and discontinuation causes for different age groups in the paediatric cohort. Also, the number and rates of adverse events are shown.

**TABLE 9:** ADR occurrence and patient incidence by organ system in the paediatric cohort.

	(0–7 months)		(8 months–1 year)		(2 – 6 years)		(7–11 years)		(12–17 years)	
	Patients with ADRs (%)	Number of ADRs (%)	Patients with ADRs (%)	Number of ADRs (%)	Patients with ADRs (%)	Number of ADRs (%)	Patients with ADRs (%)	Number of ADRs (%)	Patients with ADRs (%)	Number of ADRs (%)
	*n* = 11	*n* = 16	*n* = 19	*n* = 26	*n* = 17	*n* = 22	*n* = 32	*n* = 50	*n* = 37	*n* = 65
**Skin**	**2 (12.5)**	**2 (12.5)**	**12 (48)**	**12 (46.15)**	**8 (38.1)**	**8 (36.36)**	**19 (41.3)**	**23 (46)**	**25 (41.67)**	**26 (40)**
Exanthema (pruriginous rash)	1 (50)	1 (50)	5 (41.67)	5 (41.67)	5 (62.5)	5 (62.5)	15 (78.95)	19 (82.61)	18 (72)	19 (73.08)
Urticaria/angioedema	–	–	3 (25)	3 (25)	2 (25)	2 (25)	2 (10.53)	2 (10.53)	3 (12)	3 (11.54)
Eczema	1 (50)	1 (50)	2 (16.67)	2 (16.67)	1 (12.5)	1 (12.5)	2 (10.53)	2 (8.7)	3 (12)	3 (11.54)
Exanthema (petechiae rash)	–	–	1 (8.33)	1 (8.33)	–	–	–	–	–	–
Polymorphous erythema	–	–	1 (8.33)	1 (8.33)	–	–	–	–	–	–
DRESS	–	–	–	–	–	–	–	–	1 (4)	1 (3.85)
**Gastrointestinal**	**8 (50)**	**8 (50)**	**8 (32)**	**9 (34.62)**	**2 (9.52)**	**2 (9.09)**	**10 (21.74)**	**10 (20)**	**7 (11.67)**	**9 (13.85)**
Hepatotoxicity	3 (37.5)	3 (37.5)	3 (37.5)	3 (33.33)	1 (50)	1 (50)	2 (20)	2 (20)	3 (42.86)	3 (33.33)
Abdominal pain	2 (25)	2 (25)	–	–	1 (50)	1 (50)	5 (50)	5 (50)	2 (28.57)	2 (22.22)
Nausea and vomiting	2 (25)	2 (25)	3 (37.5)	4 (44.44)	–	–	2 (20)	2 (20)	2 (28.57)	4 (44.44)
Diarrhoea	1 (12.5)	1 (12.5)	1 (12.5)	1 (11.11)	–	–	–	–	–	–
Constipation	–	–	1 (12.5)	1 (11.11)	–	–	–	–	–	–
Spasms	–	–	–	–	–	–	1 (10)	1 (10)	–	–
**Haematological**	**6 (37.5)**	**6 (37.5)**	**5 (20)**	**5 (19.23)**	**3 (14.29)**	**4 (18.18)**	**8 (17.4)**	**8 (16)**	**7 (11.67)**	**7 (10.77)**
Leukopenia	3 (50)	3 (50)	3 (60)	3 (60)	1 (33.33)	2 (50)	4 (50)	4 (50)	3 (42.86)	3 (42.86)
Eosinophilia	3 (50)	3 (50)	1 (20)	1 (20)	2 (66.67)	2 (50)	4 (50)	4 (50)	4 (57.14)	4 (57.14)
Leukocytosis	–	–	1 (20)	1 (20)	–	–	–	–	–	–
**CNS**	**–**	**–**	**–**	**–**	**2 (9.52)**	**2 (9.09)**	**7 (15.22)**	**7 (14)**	**12 (20)**	**13 (20)**
Headache	–	–	–	–	1 (50)	1 (50)	7 (100)	7 (100)	8 (66.67)	9 (69.23)
Dizziness	–	–	–	–	–	–	–	–	4 (33.33)	4 (30.77)
Irritability	–	–	–	–	1 (50)	1 (50)	–	–	–	–
**Nutritional**	–	–	–	–	**4 (19.05)**	**4 (18.18)**	**2 (4.35)**	**2 (4)**	**2 (3.33)**	**2 (3.08)**
Hyporexia	–	–	–	–	3 (75)	3 (75)	1 (50)	1 (50)	1 (50)	1 (50)
Creatinine elevation	–	–	–	–	1 (25)	1 (25)	–	–	–	–
hypercholesterolemia	–	–	–	–	–	–	1 (50)	1 (50)	–	–
Weight loss	–	–	–	–	–	–	–	–	1 (50)	1 (50)
**Body as a whole**	–	–	–	–	**2 (9.52)**	**2 (9.09)**	–	–	**2 (3.33)**	**2 (3.08)**
Fever	–	–	–	–	2 (100)	2 (100)	–	–	–	–
Asthenia	–	–	–	–	–	–	–	–	2 (100)	2 (100)
**Musculoskeletal**	–	–	–	–	–	–	**–**	**–**	**3 (5)**	**3 (4.62)**
Myalgias	–	–	–	–	–	–	–	–	3 (100)	3 (100)
**Sensory organ**	–	–	–	–	**–**	**–**	**–**	**–**	**2 (3.33)**	**2 (3.08)**
Blurry vision	–	–	–	–	–	–	–	–	2 (100)	2
**Other**	**–**	**–**	**–**	**–**	**–**	**–**	**–**	**–**	**1 (1.67)**	**1 (1.54)**
Neuropathic pain in hands and feet	–	–	–	–	–	–	–	–	1 (100)	1 (100)
**Total**	**16 (100)**	**16 (100)**	**25 (100)**	**26 (100)**	**21 (100)**	**22 (100)**	**46 (100)**	**50 (100)**	**60 (100)**	**65 (100)**

*Note*: Detailed description of the 179 ADRs occurring in the 116 paediatric patients segregated by organ system. For each age group, the first column, *Patients with ADRs*, displays the number of patients presenting with at least one ADR and its corresponding percentage. The second column, *Number of ADRs*, describes the observed number of ADRs and its corresponding percentage.

A positive association between the severity of BZ‐related ADRs and treatment discontinuation was found in both children and adults. In children, 52.5% (21/40) of discontinuations were observed in the 40 patients with moderate/severe ADRs compared to 14.4% (13/90) of discontinuations in the 90 patients with mild ADRs (OR = 0.16, 95% CI = 0.06–0.4, *P* < .0001), while in adults, 81.81% (9/11) of discontinuations in the 11 patients with moderate/severe ADRs compared to 4.35% (1/23) of discontinuations in the 23 patients with mild ADRs was observed (OR = 0.01, 95% CI = 0–0.1, *P* < .0001).

### 3.4 Treatment completion

Most patients, 89.77% (465/518), completed treatment. Although there was a significant difference between children 91.76% (412/449) and adults, 76.81% (53/69) (OR = 3.36, 95% CI = 1.71–6.4; *P* < .001; Table 6). Treatment discontinuations were related to ADRs in 56.6% (30/53) of patients (Table 7). There was no significant difference between children and adults. Notably, 83.3% (25/30) of ADR‐related discontinuations were skin ADRs. In total, 10.61% (55/518) of patients temporarily interrupted BZ. Of these subjects, 11.35% (51/449) were children and 5.8% (4/69) were adults, with a median temporary interruption length of 7 days (IQR_25–75_ = 3–12 days for children; 4–10.25 days for adults). In 40 patients (38/51 children and 2/4 adults), this temporary interruption was due to ADRs, while two patients interrupted by their own decision, eight because they ran out of BZ and five for unknown reasons.

### 3.5 Paediatric cohort analysis

In children, a treatment completion rate greater than 84% was observed, with differences between age groups (Table 8). A higher incidence of ADRs and temporary interruptions was evident from age 7 and onwards (Table 9). In children below the age of 8 months, ADRs most commonly affected the gastrointestinal system: 72.72% (8/11). Conversely, the skin became the predominant system affected in older age groups: 63.15% (12/19) in children between 8 months and 2 years, 47.05% (8/17) in those aged 2–7 years, 59.37% (19/32) in the 7–12 years age group, and 67.56% (25/37) in children between 12 and 18 years. A detailed description of paediatric ADRs by age group can be found in Table 9.

## 4 DISCUSSION

WHO and PAHO have published guidelines stating that all patients with acute ChD and most patients with chronic ChD are eligible for antiparasitic treatment with either BZ or NFX.^7^, ^13^ However, the relatively long treatment periods required, unclear efficacy rates in some geographic regions, poorly studied PKPD profiles and the reported high incidence of adverse events still raises concerns among physicians and patients about how extensive BZ and NFX use should be. Since BZ is most frequently prescribed, understanding its adverse effects can assist medical providers to devise specific interventions to improve patient care.

We present the results of a large retrospective cohort study of adult and paediatric ChD patients treated with BZ. The majority of individuals in this cohort were children, with most adult patients being mothers of these children. The Hospital de Niños “Ricardo Gutiérrez” serves as a national reference centre dedicated to the care of newborns and paediatric patients with ChD in Buenos Aires city, Argentina, an area devoid of vectorial transmission. Consequently, the majority of children in our study were congenitally infected with ChD. While only 20.28% of adult patients were confirmed as congenital cases, this proportion could be higher due to the challenges associated with retrospectively diagnosing congenital infections in patients from endemic areas.

Similar to previous reports,^14^, ^15^, ^16^, ^17^ most of the BZ‐related ADRs in our cohort were mild or moderate and resolved without sequelae. Treatment discontinuation rates in this study were high in adults: 23.18% (16/69), but low in children: 8.24% (37/449). Nevertheless, the overall incidence of BZ‐related ADRs was low 29.34% (152/518), mostly due to a low incidence of ADRs in children of 25.83% (116/449). A recent systematic review reported a slightly higher BZ‐related ADR rate of 44.1%.^4^ The median time to appearance of ADRs in our cohort was 1 day earlier in adults than in children (8 *vs*. 9 days). Most ADRs occurred within the first month of treatment for both groups, suggesting that most BZ‐related ADRs are not dependent on cumulative doses.

While only 10% of patients discontinued treatment, a positive association was observed between the severity of BZ‐related ADRs and treatment discontinuation in both adults and children. This suggests that, although moderate and severe ADRs are less frequent than mild ADRs, they still significantly contribute to a treatment adherence rates.

Aligning with other publications,^4^, ^9^, ^16^, ^17^, ^18^ ADRs in this study most commonly involved the skin and mucous membranes. Exanthema was the most frequent skin ADR, followed by urticaria/angioedema and eczema. Most rashes appeared around the ninth day of treatment, as is commonly described for cutaneous drug reactions associated with other antimicrobials, such as fluoroquinolones, and some anticonvulsants. There was a single case of DRESS in this study, an ADR of rare occurrence in the context of trypanocidal treatment (less than 1% reported^19^). It commonly presents with a morbilliform cutaneous eruption along with fever and lymphadenopathy.^20^ The severity of this syndrome correlates with its systemic impact, which can result in multi‐organ failure. As occurred in this case, the most important step in the management of DRESS is early diagnosis and immediate cessation of the suspected offending drug and treatment with an immunosuppressive agent if required.^21^

As has been previously reported,^9^, ^10^, ^22^ we also found that ADRs were mild in children and in most cases did not require treatment interruption. The occurrence of ADRs and BZ‐related ADRs were significantly more frequent in adults. Although not a complete explanation for the contrasting incidence of ADRs between adults and children, a number of age‐differentiating pharmacological aspects could help in understanding this difference. In previous studies,^10^, ^23^ we found that children treated with the same dose per kg as adults achieved lower BZ concentrations in blood, suggesting a faster clearance of the drug in paediatric patients. BZ is metabolized primarily in the liver, and, like many other drugs metabolized this way,^24^, ^25^ it would be expected to undergo faster liver clearance in children over 2 years old, compared to adults, potentially leading to shorter half‐lives and lower steady‐state plasma concentrations. Moreover, a recent exploration of BZ metabolism revealed metabolites and, glucuronate and glutathione conjugates with potentially toxic structures. This may help to partially the explain the differential incidence in treatment adverse events (e.g., if children had higher elimination capacity for those toxic metabolites, or had differential metabolic routes to eliminate the drug).^26^

The other drug available to treat ChD, NFX, exhibits a distinct ADR profile, most commonly causing anorexia along with other gastrointestinal side effects whilst also affecting the CNS.^5^ The differences in ADR profiles between both drugs are not clearly explained to date. Despite both drugs belonging to the same chemical group (nitro‐drugs), there is limited research on their metabolism and metabolite profiles.^27^, ^28^ This knowledge gap poses a significant obstacle to formulating conjectures about the pharmacological reasons behind these ADR differences.

The primary limitation of this study is its retrospective nature, conducted over an extended period. There is an inherent risk of bias due to the limited availability of detailed information on the incidence or severity of ADRs. Additionally, the outdated technology used in data collection during the early years may result in a lack of detail in certain instances. But despite these limitations, we believe these results contribute valuable information to the scarce existing evidence on this topic.

BZ is the most widely used of only two drugs available to treat ChD. A more extensive understanding of its toxicity profile will help medical practitioners to use it more safely in both paediatric and adult ChD patients. There are few certainties in the field of ChD; however, it is well known that most primary infections occur during childhood. Thus, early diagnosis and treatment in children is crucial to prevent long‐term ChD sequelae.^29^

## 5 CONCLUSION

Our study found that most ADRs were mild to moderate and severe ADRs were rare. While optimized dosing schedules and new drugs are needed, avoiding BZ exclusively due to safety concerns is not supported by the evidence. Appropriate monitoring and interventions to manage ADRs effectively are strongly recommended.

## CONFLICTS OF INTERESTS STATEMENT

None of the authors have conflict of interests to declare.

## Data Availability

This research was funded in part, by the Wellcome Trust [222 754/Z/21/Z]. For the purpose of Open Access, the author has applied a CC BY public copyright licence to any Author Accepted Manuscript version arising from this submission.
